# Fine-scale differences in diel activity among nocturnal freshwater planarias (Platyhelminthes: Tricladida)

**DOI:** 10.1186/1740-3391-9-2

**Published:** 2011-04-10

**Authors:** Paola Lombardo, Marco Giustini, Francesco Paolo Miccoli, Bruno Cicolani

**Affiliations:** 1 Department of Environmental Sciences - "Marco Giustini" Ecology Lab, Coppito Science Center, University of L'Aquila, I-67100 L'Aquila, Italy

## Abstract

**Background:**

Although most freshwater planarias are well known photonegative organisms, their diel rhythms have never been quantified. Differences in daily activity rhythms may be particularly important for temperate-climate, freshwater planarias, which tend to overlap considerably in spatial distribution and trophic requirements.

**Methods:**

Activity of stress-free, individually tested young adults of three common planarian species was recorded at 3-h intervals in a 10-d experiment under natural sunlight and photoperiod during autumnal equinox (D:L ~12:12). Individual activity status was averaged over the 10-d experiment, each tested individual thus serving as a true replicate. Twelve individuals per species were tested. Food was provided every 36 h, resulting in alternating day- and nighttime feeding events. Activity during the first post-feeding h was recorded and analyzed separately. Statistical procedures included ANOVAs, correlations, and second-order analyses of angles.

**Results:**

*Dugesia (= Girardia) tigrina *Girard 1850 exhibited clear nocturnal behavior, *Dugesia (= Schmidtea) polychroa *Schmidt 1861 was predominantly but not exclusively nocturnal, and *Polycelis tenuis *Ijima 1884 was relatively more active from midnight through noon. Species-specific activity peaks were statistically similar, with peaks at dawn for *P. tenuis *and just before midnight for the two dugesiids; however, *D. tigrina *was comparatively more active in the early night hours, while *D. polychroa *was more active than *D. tigrina *during daytime. *D. tigrina *also responded less readily to daytime food addition. *P. tenuis *remained poorly active and unresponsive throughout the experiment. Individual variability in diel behavior was highest for *D. polychroa *and lowest for *D. tigrina. P. tenuis*'s general low degree of activity and late activity peak in the experiment may be related to a strong reliance on external stimuli.

**Conclusions:**

The tested species are mainly nocturnal, consistent with their photonegative characteristics. The fine-scale differences in diel behavior among these three triclad species may not be sufficient to allow coexistence in the wild, with the nonnative *D. tigrina *eventually displacing *D. polychroa *and *P. tenuis *in many European waters. The link between planarian diel rhythms and ecological characteristics are worth of further, detailed investigation.

## Background

The photonegative behavior of most freshwater planarias was consistently observed by early naturalists and ecologists [1-3]. Subsequent, more quantitative studies confirmed these early observations [e.g., [4,5]]. Today, planarian photonegative behavior is a synonym for nocturnal habits, and is used as the basis for ecophysiological exercises in textbooks, laboratory manuals, and in pharmacological and medical tests [e.g., [6]].

A few isolated observations on dugesiid planarias under natural photoperiod suggest that responsiveness to stimuli follow daily cycles, with lower responsiveness in the afternoon and early evening [7,8]. However, the vast majority of published investigations on planarian phototaxis have employed observations of planarian response to abrupt, artificial exposure to light, often with simple light-*vs*.-dark conditions [e.g., [3,5,6]]. Such an "all-or-nothing" approach did not allow to ascertain planarian behavior in transitional light such as dawn or dusk. With very few exceptions [7,8], observations were explicitly or implicitly carried out during daytime, i.e., at a time convenient for the investigators, despite the known (albeit short-lived) habituation to light conditions for some planarias [e.g., [9]]. More recent findings of diel cycles in planarian melatonin production or storage [10,11] also cast doubts on the validity of such artificial dark-*vs*.-light observations as evidence for nocturnal behavior. Therefore, the aversion to light by planarias in the early studies cannot be ascribed positively to inherent nocturnal habits.

In order to test the hypothesis that planarias are really nocturnal animals as the behavioral literature suggests, we have determined the diel activity patterns for three species of freshwater planarias common in lake littoral habitats of central Italy, under stress-free, natural-light conditions. The statistical null hypothesis (H_0_) that the activity of planarian species does not change in a 24-h period was tested with a combination of parametric ANOVAs and second-order analyses of angles. The same approach was used to investigate planarian response to alternating daytime and nighttime food inputs using a separate dataset.

A quantitative study addressing the diel habits of freshwater planarias is much needed not only *per se*, but also to help explain the ecology of planarias and of benthic aquatic communities at large. In fact, daily rhythms in many aquatic organisms, including the drift of stream insects [12,13] and vertical or horizontal migration of zooplankton in lakes [e.g., [14]], are often closely associated with interspecific and community dynamics, usually as a strategy to avoid predation [e.g., [14,15]]. Because our experimental species are strongly regulated by intra- and interspecific competition [16-19], highly overlap in trophic [16,17,20,21] and habitat requirements [22-24] and in geographical distribution in Europe [22,25-28], we further hypothesized that differences in their diel activity rhythms could reduce interspecific competition and allow coexistence.

However, large-scale distribution observations are not supported at the local scale, as planarian assemblages are typically dominated by one or two species, and common planarian species are rarely found coexisting in high numbers at the habitat scale [[19,20,23,24,27-29]; authors' personal observation], suggesting that differences in daily activity rhythms (if any) may not be sufficient to separate freshwater planarias ecologically. Despite their potential importance in explaining the discrepancy between the highly overlapping geographical distributions and mutual exclusion at the local scale, temporal aspects of freshwater planarian ecology remain typically overlooked. The results on the basic daily rhythms of common planarias thus were also integrated with information from the literature to discuss freshwater planarian ecology, with an emphasis on a possible link between circadian rhythms and interspecific interactions.

## Methods

### Study organisms

The three species of planarias investigated (Table 1) are common in a variety of waterbodies throughout Italy and much of Europe [22,23,25]. *Dugesia (= Girardia) tigrina *Girard 1850 is a North American native that was first recorded in Europe in 1925 [30], while the European native *Dugesia (= Schmidtea) polychroa *Schmidt 1861 has been introduced into North America in the late 1960s [31,32]. *Polycelis tenuis *Ijima 1884 is common and widespread through much of its native Europe [22]. All species are predominantly predators on small invertebrates and include gastropods in their diets [17,20,29,33]; all may additionally act as scavengers on carryon or recently dead organic matter [20,21,34-36]. All species are hermaphroditic, are adapted to warm, hard, and moderately eutrophic waters [[22,36-39]; authors' personal observations], and tend to be abundant when present [[18-22,29,40]; authors' personal observations]. Species identification was based on morphological traits and squash mounts of live individuals using [22]. Nomenclature follows [22] and [25].

**Table 1:** Description of tested planarias

		**body length at t**_**0 **_**(in mm)**	
		
species	family	range	average ± std error
*Dugesia (= Schmidtea) polychroa*	Dugesiidae	5.5-10.0	7.8 ± 0.4
*Dugesia (= Girardia) tigrina*	Dugesiidae	5.2-9.5	6.7 ± 0.3
*Polycelis tenuis*	Planariidae	5.2-8.1	6.4 ± 0.2

The three species of planarias investigated, listed alphabetically. All species belong to the infraorder Paludicola (free-living freshwater planarias). Body length refers to the individuals used in the experiment; *n *= 12 for all.

Experimental planarias were randomly picked from laboratory cultures comprising individuals collected in late summer 2008, at a time when populations were dominated by small-sized individuals (Table 1), as is typical of these species [e.g., [29]]. *D. polychroa *and *P. tenuis *naturally co-occurred at a vegetation-devoid gravel-bottom site (42°32' N, 12°44' E; WGS 84 coordinates) along the northern shore near the western tip of Lake Piediluco. *D. polychroa *and, to a lesser extent, *P. tenuis *were the most common species of an abundant *in situ *triclad community that comprised also *D. lugubris *Schmidt 1861 and *Dendrocoelum lacteum *O.F.Müller 1774. *D. tigrina *was the only triclad at a richly vegetated, clayey-bottom site (42°17' N, 13°33' E) in Lake Sinizzo. All species were abundant *in situ *from early spring through late autumn (March-November). Source lakes are hardwater and meso-eutrophic [[41]; authors' unpublished data]. Lake Piediluco is located ~75 km NNE of Rome within the River Tiber watershed, and Lake Sinizzo is located ~15 km ESE of the city of L'Aquila in the River Aterno watershed. Both collection sites are open-canopy, shallow (~0.5 m), with clear water and a rich benthic invertebrate fauna.

Planarias were maintained in shallow-water, predator-free containers with lake water, coarse-gravel substratum, macrophyte fragments, and a variety of substratum-associated, potential micro- and macroinvertebrate prey, all coming from the source lakes. Material from different lakes was kept in separate aquaria. The original lake water was gradually diluted and eventually replaced with tap water over a few weeks. Water was kept aerated by means of an aquarium air pump and refreshed every week. The natural diet of cultured planarias was integrated with commercially available, protein-rich food for aquarium cichlid fishes in pellets (diameter ~2.5 mm; thickness ~0.75 mm), which planarias were able to detect and consume within a few minutes from addition. Planarias were maintained outdoors in a patio area in suburban Rome, Italy (41°43' N, 12°21' E), protected from direct sunlight, rain, and prevailing winds, so that culturing conditions (including light irradiance and photoperiod) followed natural conditions but with dampened short-term fluctuations. Cultured populations remained abundant and healthy with sustained reproduction through and beyond the experiment period.

### Experimental setup

The experiment was carried out alongside the culturing aquaria adapting the methods in [42] for a similar-purpose experiment with gastropods. Thirtysix analytically clean clear-glass jars were each filled with 100 mL of tap water and placed in 3 rows × 12 columns on a white-surface desk. Based on qualitative observations in culturing aquaria, a clean, small (diameter ~2-3 cm) cobble was added to each jar to provide a shelter for planarias when inactive. Jars received indirect, diffuse natural daylight from dawn through dusk (SSE through WNW exposure). Midday light irradiance at the jar water surface was ~50-60 μmol m^-2 ^s^-1^, simulating natural conditions in shaded, shallow-water lake littoral zones (P. Lombardo, unpublished data). Jars were left undisturbed for ~12 h to lose excess chlorine from water; equilibrium with ambient temperature was reached at ~22:00.

Twelve typically pigmented, representative-sized adult individuals of each species (Table 1) were randomly picked up ~2 h before the beginning of the experiment and transferred into the experimental jars (one per jar) following a modified Latin-square layout, in which each three-jar column was assigned randomly within each of four contiguous Latin squares, so that each square of 3 × 3 jars featured one individual of each taxon per row and per column. Such a layout allowed to distribute any small between-row difference in light conditions equally across species. Planarian body size was recorded at the beginning of the investigation (t_0_) as total length (head to tail) on actively gliding individuals in clear-glass Petri dishes on a graduated paper sheet (grid resolution = 0.5 mm) [19,29,43].

Because of an apparent "all-or-nothing" behavior displayed by planarias, the behavioral gradient used in [42] was not applicable. Each planarian individual was recorded simply as active or inactive every 3 h starting from 0:00 (midnight) on 4 September (i.e., ~2 h after planarian addition to jars) through 21:00 on 13 September 2008, spanning ten consecutive 24-h cycles. Inactivity was defined as absence of any detectable body movement during 10-15 s of close visual inspection. Inactive individuals were often found adhering to the substratum with their body partially contracted. Preliminary observations showed that planarias not visible from the exposed sides of the jars (i.e., from the top and the sides) were resting under the cobble; such individuals therefore were recorded as inactive during the experiment, avoiding any physical contact by the investigator that may have startled the planarias and altered their behavior. (Planarias of all species were very sensitive to artificially induced water movements in culturing aquaria and in preliminary trials.)

Nighttime observations were made with a small flashlight covered with a dark-red semitransparent plastic filter to minimize disturbance [e.g., [44]]. When activity mode could not be discerned at first glance, the flashlight beam was directed away from yet-to-be-observed individuals to avoid artificial alterations in activity. Inactive planarias that were disturbed during observation rounds (day- or nighttime) regained their original inactive mode within a few minutes, so the mild (if any) disturbance brought about by the investigator did not alter results at subsequent observations. Individual activity bouts also were much shorter (typically a few to ~30-40 min) than the 3-h observation intervals, so that activity records at subsequent observation times were deemed sufficiently separated and independent. Records for the one individual that died during the investigation were excluded since time of death, as death appeared accidental (desiccation following entrapment in the calcareous formation at the water edge at d_8_; d_1 _= t_0_) with no behavioral or physical alterations until the last observation before death. This individual was thus maintained as a replicate, but its activity data were averaged over a lower number of daily cycles.

Each round of observations began with recording planarian activity, followed by determinations of water temperature and pH, and light irradiance at the jar water surface. Physicochemical variables were determined as in [42]. Water temperature and pH remained within the relatively narrow ranges of ~20-26°C (night-day) and 8.4-8.6 units, respectively. Such experimental ranges are well within the tolerance ranges of the species investigated [[22]; authors' personal observations]. Temperature and pH also were not correlated with planarian activity (*r*^2 ^= 0.08-0.36 and *p *= 0.12-0.51 for linear correlations for each species with *df *= 6), and are not treated further. Light:dark conditions followed the natural daylight cycle, around autumn equinox (D:L ~12:12 h), with the 6:00 and the 18:00 observation rounds corresponding to dawn and dusk conditions, respectively. Each complete round of observations and measurements was carried out in ~8-10 minutes.

Food was added at regular 36-h intervals since 13:00 on d_2_. The 36-h interval allowed to have alternating daytime and nighttime food additions, thus avoiding food-induced bias in diel activity patterns. Food consisted in one fish food pellet as described earlier, and was removed after 7 h from addition to avoid excess leftover that may have led to bacterial development in the jars, and to stimulate planarian response to the next feeding event. The 7-h hiatus was based on preliminary observations, during which planarias appeared satiated and seldom returned to feed on the pellet by the second-next "regular" observation round. Pellet leftovers were removed at the end of such second-next "regular" observation round with small, nonintrusive Pasteur pipettes. Response to food inputs was determined as changes in activity at 5-min intervals from just before food addition (at 13:00 or 1:00) for the first 30 minutes and again as a one-time observation 1 h after food addition. The 1-h food-addition events were thus carried out halfway through two "regular" observation rounds, minimizing disturbance that could have otherwise affected planarian behavior. Food addition did not cause appreciable alterations in pH.

The experiment was managed with an ethical approach, including a humane treatment of experimental animals, which were returned unharmed to the culturing aquaria after the experiment. *In situ *collection sites for the experimental planarias were neither protected nor contaminated. The article reports an original experimental idea and original data. All the data used in the article are the result of direct observation, and no outliers have been discarded. The research has been approved by the Head of the Department of Environmental Sciences of the University of L'Aquila.

### Statistical analysis

Taxon-specific analysis was based on the times of active or inactive occurrences of each planarian individual averaged over the 10-d experimental duration, obtaining a single value per individual [42]. The same approach was applied to food addition data, analyzed separately. The 12 individuals per species were thus true replicates, and one-way, type I ANOVAs followed by Student-Newman-Keuls (SNK) multiple-comparison tests (*p *≤ 0.05) were used to detect differences among observation times. Data were expressed as percent of total number of individuals, so transformation was not necessary [45]. ANOVA- and SNK-based differences were considered significant at *p *≤ 0.05.

Species-specific peak activity times were calculated as average angles on angle-transformed hourly data [] with associated coefficients of angular concentration (*r*_c_) [45-47]; differences were tested with a second-order analysis of angles [48] as modified in [49]. The angular concentration (*r*_c_) is a measure of species-specific variability in behavioral activity, ranging from zero (maximum variability) to one (absence of individual variability). Angular statistics were not suited to incomplete-cycle food addition data and were applied only to complete 24-h cycle data. Graphical rendition of diel data was circular [45] unless clarity became an issue; linear rendition was adopted in such cases.

Temporal changes in light irradiance were detected with a one-way, type I ANOVA followed by an SNK test (*p *≤ 0.05) on log-transformed data [Bartlett's formulation: *x*' = log_10 _(*x *+ 1)]. Correlations between selected datasets used untransformed data because of analysis reliability when nonnormality is not extreme [45]. Correlations used activity data from 3-h-spaced observation rounds because activity bouts were typically much shorter than 3 h, so that independence of data could be safely assumed. Correlations were not performed on food-event data because of the evident autocorrelation between the 5-min-spaced observations. All times were corrected for daylight saving time and are reported as standard CET (Central European Time).

## Results

**Figure 1:**
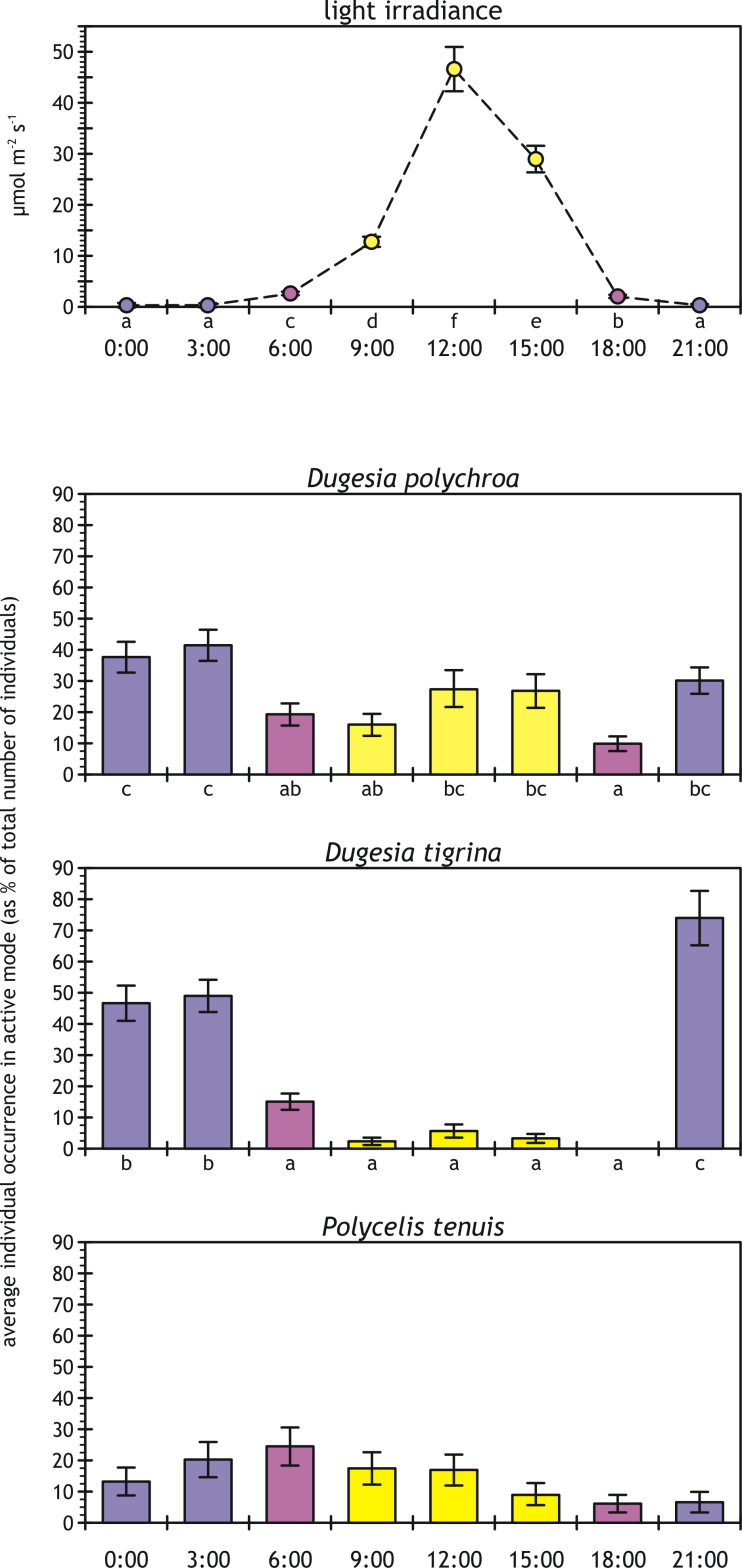
**Diel cycles in light irradiance and planarian activity**. Light irradiance (top panel; average ± standard error; *n *= 10 for each time period) and average planarian individual activity (bottom three panels; average ± standard error; *n *= 12 for each time period) during the 24-h observation cycles, with observations carried out every 3 h starting at midnight on d_1_. Full daylight times are in yellow, nighttime hours in blue, and twilight hours in purple. Lower-case letters identify significantly different average values according to SNK tests (*p *≤ 0.05) performed after significant one-way, type I ANOVAs on original (*F*_*D.polychroa *_= 5.746, *p *< 0.001; *F*_*D.tigrina *_= 42.766, *p *< 0.001; *F*_*P.tenuis *_= 2.041, *p *= 0.06; *df *= 7,88 for all) or log-transformed data (*F*_light _= 278.783, *p *< 0.001, *df *= 7,72).

Light irradiance at the water surface exhibited evident day-night cycles (Figure 1, top panel). Weather conditions were variable but overall benign, without overcast or rainy days, resulting in complete statistical separation of daytime light conditions from dawn through dusk (included).

Though most planarian individuals were active at night, only *D. tigrina *exhibited evident nocturnal habits (Figure 1; Table 2). *D. polychroa *exhibited predominant but not exclusive nocturnal habits, with the ~40% peaks in hourly activity at 0:00 and 3:00 only incompletely separated from the daytime average activity at 12:00 and 15:00 (SNK test in Figure 1). Activity patterns for *D. polychroa *also were not associated with diel light conditions (Table 2). The degree of activity for the two dugesiids was statistically similar at 0:00 and 3:00; *D. tigrina *was more active than *D. polychroa *at 21:00, and *D. polychroa *was more active than *D. tigrina *from 9:00 through 18:00 (Table 3). Though differences remained statistically blurred at best, daily minima in activity for all species were at dusk; *D. tigrina *was never found active at this time (Figure 1).

**Table 2:** Relationship between light and activity

species	***r***^***2***^	*p*	type	trend
*D. polychroa*	0.005	0.87	lin	-
*D. tigrina*	0.653	<0.01	log	-
*P. tenuis*	0.0003	0.97	lin	+

Correlations between diel light irradiance and planarian activity, using the values reported in Figure 1 (*n *= 8 and *df *= 6 for each correlation). Best fitting correlations are reported for each species; lin = linear and log = logarithmic relationships; positive and negative trends are reported as "+" and "-", respectively.

**Table 3:** Across-species differences in activity

time of observation	ANOVA		SNK separation		
	*F*	*p*	*D. polychroa*	*D. tigrina*	*P. tenuis*
0:00	7.665	<0.01	b	b	a
3:00	5.499	<0.01	b	b	a
6:00	0.790	0.51	- - - test not performed - - -		
9:00	3.471	0.03	b	a	b
12:00	3.702	0.02	b	a	ab
15:00	6.920	<0.01	b	a	a
18:00	3.630	0.02	b	a	ab
21:00	22.883	<0.01	b	c	a

Across-species differences in activity (based on the data presented in Figure 1) according to one-way ANOVAs (*df *= 3,33 for all) coupled with SNK tests at *p *≤ 0.05. Different letters identify SNK-based statistically different average activity, listed alphabetically (a = lowest value).

*P. tenuis *was the least active of the three species (Figure 1 and Table 3), with a maximum of 24.3% of the experimental group of individuals active at 6:00. However, activity of *P. tenuis *remained marginal, with nonsignificant differences in the degree of activity across a 24-h cycle (Figure 1); activity also was not correlated with diel light conditions (Table 2).

**Figure 2:**
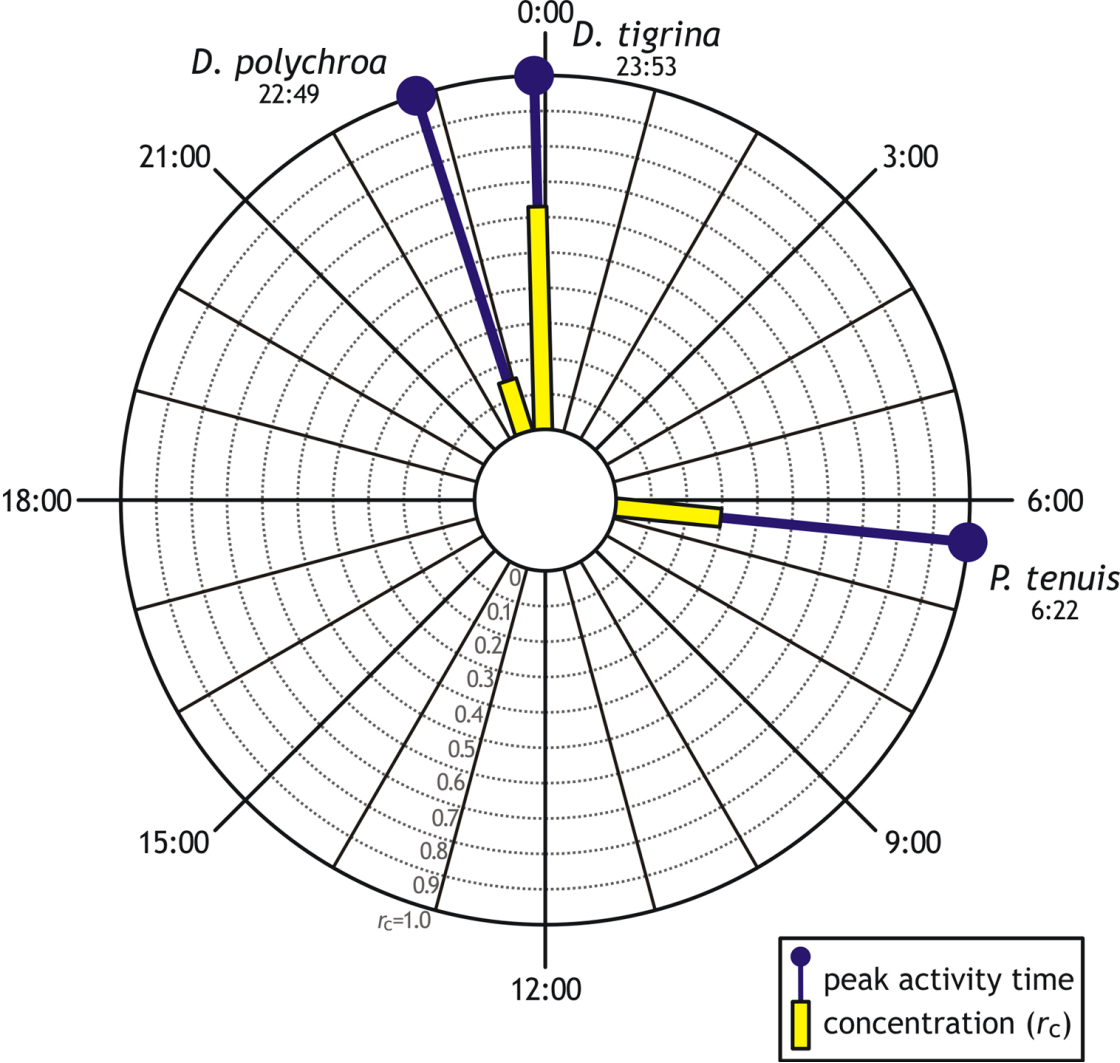
**Daily peaks in planarian activity**. Daily peak activity times for the three species examined, calculated as average angular-transformed hourly data. The angular concentration (*r*_c_), an inverse measure of individual variability, also is given. Pooled standard error, used to separate significantly different averages [49], was not calculated because species-specific daily peak activity times were not statistically separated (second-order Hotelling test: *F *= 1.407, *p *= 0.185).

The coefficient of angular concentration was relatively high for *D. tigrina *(*r*_c _= 0.63), and low for *P. tenuis *(*r*_c _= 0.30) and especially for *D. polychroa *(*r*_c _= 0.15) (Figure 2). Daily activity peaks were statistically similar for the three species (nonsignificant Hotelling test in Figure 2). (Angular) average daily peak activity time for the three species collectively considered was 23:20.

**Figure 3:**
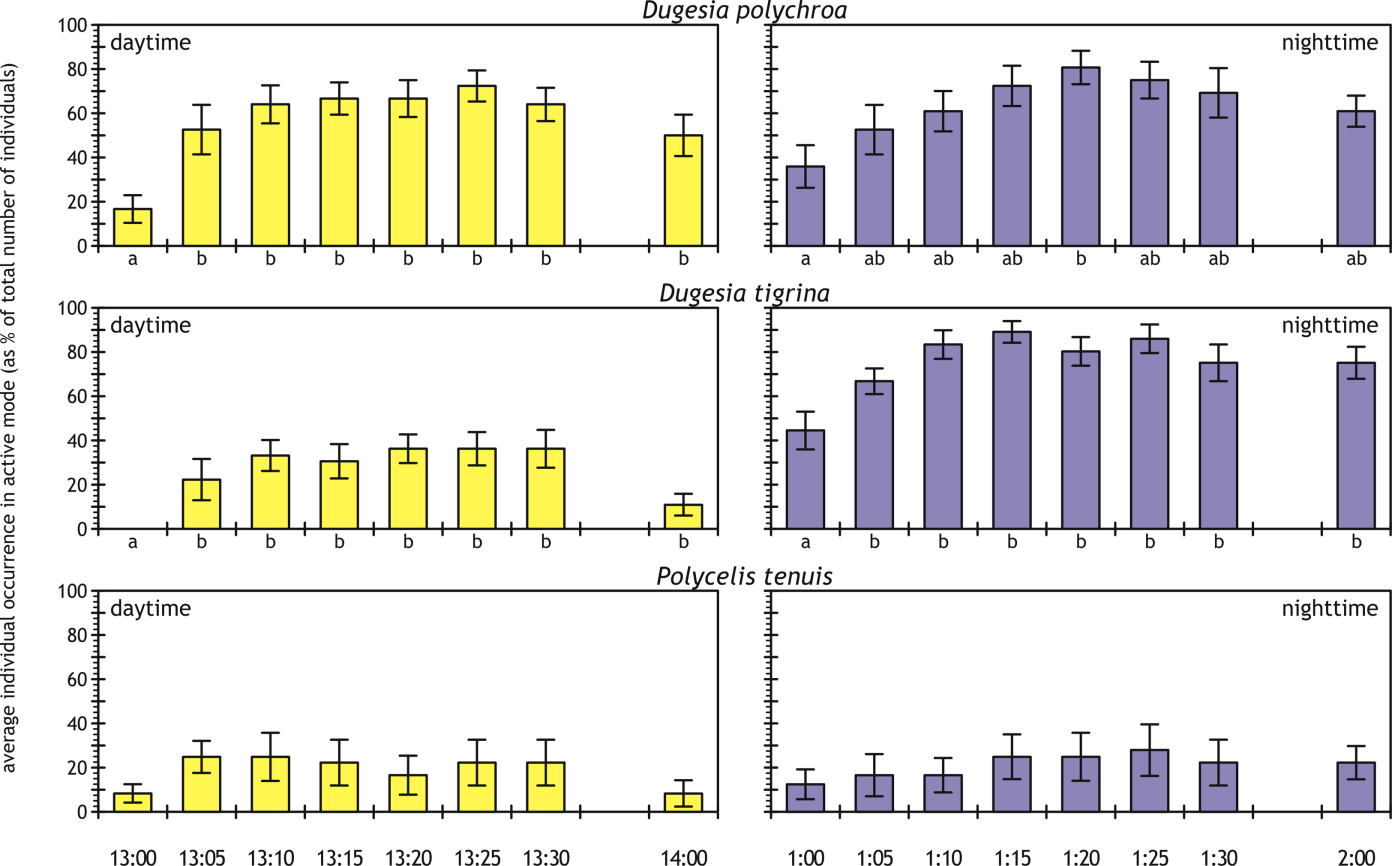
**Planarian activity following food inputs**. Occurrence in active mode (as % of total number of individuals; average ± standard error) just before (13:00 or 1:00), at 5-min intervals for the first 30 min, and 1 h after daytime (left panels, in yellow) and nighttime food addition (right panels, in blue), for the three species examined. Lower-case letters identify significantly different average values according to SNK tests (*p *≤ 0.05) performed after significant one-way, type I ANOVAs (*D. polychroa: F*_day _= 4.509, *p *< 0.001; *F*_night _= 2.368, *p *= 0.02; *D. tigrina: F*_day _= 3.734, *p *< 0.01; *F*_night _= 4.316, *p *< 0.001; *P. tenuis: F*_day _= 0.616, *p *= 0.74; *F*_night _= 0.304, *p *= 0.95; *df *= 7,88 for all).

Food addition was associated with an increase in the level of activity for the two dugesiids, but not for *P. tenuis *(SNK separation in Figure 3). Significantly more dugesiid individuals had become active than inactive by 5-15 minutes after nighttime food addition (paired activity-*vs*.-inactivity *t*-tests [50]; results not shown). Daytime food addition was associated with a significant increase in activity only for *D. polychroa*, while *D. tigrina *remained significantly inactive as a population (paired *t*-tests; results not shown). *P. tenuis *remained significantly inactive throughout the food addition events regardless of time of day, while *D. polychroa *and *D. tigrina *remained significantly more active than pre-feeding conditions 1 h after food addition (SNK separation in Figure 3; incomplete for *D. polychroa *at night).

## Discussion

### Diel activity patterns and response to food

Activity was generally nocturnal for all species (Figures 1 and 2), supporting earlier findings of strong photonegative behavior for dugesiids and other triclads [[3,5,36]; authors' personal observations]. However, only *D. tigrina *exhibited clear nocturnal habits (Figures 1 and 2; Table 2), while *D. polychroa *was active virtually throughout a 24-h cycle (Figure 1), with high individual variability in activity behavior (low *r*_c _coefficient in Figure 2). Our results support earlier findings of aversion to light by *D. tigrina *stronger than for other dugesiids [5], and are consistent with the active seek-out hunting strategy displayed by *D. polychroa *[e.g., [35]]. The low interindividual variability for *D. tigrina *(high *r*_c _value in Figure 2) may be related to the highly gregarious behavior of this species [[51]; authors' personal observations]. The high individual variability of *D. polychroa *is consistent with its individualistic behavior, with typical one-on-one prey seeking, chasing, and capture, though several individuals may accumulate on a single subdued prey after capture (authors' personal observations). Dusk was the moment of lowest activity for all species, quantitatively or qualitatively (Figure 1), suggesting that planarias use dusk hours to rest before entering their diel activity peaks at night. Similar late-afternoon minima in responsiveness to stimuli were found in earlier behavioral studies for *D. tigrina *[7] and *D. dorotocephala *[8].

Though the general daily patterns remained consistent with a night-through-midday maximum responsiveness for freshwater planarias [7,8], *P. tenuis *exhibited activity patterns and general behavior different from the two dugesiids (Figures 1 through 3). *P. tenuis *is inherently less active or responsive than *D. tigrina *[24]. However, the overall very low degree of activity (Figure 1) and poor response to food inputs (Figure 3) are in striking contrast with *P. tenuis*'s active behavior and high responsiveness to the very same food supplied in the culturing aquaria, as well as with the highly active, seek-out hunting strategy displayed in other investigations [e.g., [17,35]].

The unresponsive behavior by experimental *P. tenuis *may be related to the planarias having been individually tested in isolation. In fact, chemoreception is thought to be the main sensory mechanisms by which freshwater planarias interact with one another and with their potential prey and predators [35,52]. We have also found all planarias in culturing aquaria very responsive to small water movements at any time of the day, suggesting a nontrivial additional role of mechanoreception in planarian behavior, as found elsewhere [e.g., [24,36] and references therein]. Thus, *P. tenuis *may rely heavily on chemical and/or mechanical cues from co-occurring con- and/or allospecifics, which would signal a feeding opportunity, rather than being directly stimulated by food "odors" (at least for the artificial food in our experiment and cultures). *P. tenuis *is often found co-occurring with *D. polychroa *(as at our collection site in Lake Piediluco) and/or with the closely related *D. lugubris *[e.g., [16,23,53]], supporting the view that chemical and/or mechanical cues from coexisting native planarias provide *P. tenuis *with a gain that offsets potential competition [[54], but see [53]]. *P. tenuis*'s relative peak in diel activity at dawn (Figures 2 and 3) thus may be an experimental artifact, with hungry planarias eventually venturing on their own after an entire night spent waiting for some chemical and/or mechanical cue that never materialized because of the isolated condition. *D. polychroa *and *D. tigrina *instead may rely on such cues less extensively than *P. tenuis*.

### Ecological implications: Potential influence of differences in diel activity cycles on predation, competition, and coexistence

The overall low degree of diel activity (Figure 1) but quick response to pulse food inputs (Figure 3) suggest that planarias tend to optimize their energy expenditures by concentrating foraging activities either during limited times of the day, or as a response to external stimuli. Such an energy-saving foraging behavior is often adopted by predators [e.g., [35,55]], and may alternatively or additionally lower the risk of predation, as planarias tend to hide under cobbles and in other difficult-to-reach spaces when inactive (authors' personal observations). Under this light, the day-long active *D. polychroa *may be more vulnerable to predation than the more strictly nocturnal *D. tigrina*.

Temporal partitioning may contribute to alleviate competitive and predator-prey interactions by decreasing the chance of encounters between potentially interacting species [e.g., [56]]. Unfortunately, too little is known about the daily rhythms of other invertebrates that may compete, prey on, or be preyed upon by lake planarias to allow a meaningful discussion of the community-scale implications of planarian (mainly) nocturnal habits. However, littoral gastropods, which constitute a refuge trophic resource for dugesiid planarias [e.g., [16,20]], appear to be mostly diurnal [42], suggesting that predation pressure on snails by dugesiid planarias, often high in laboratory settings [e.g., [43]], may not be as high under natural conditions because of temporal partitioning. Under this light, the highest temporal overlap and hence highest potential for interaction is between the predominantly nocturnal but day-long active *D. polychroa *(Figure 1) and the predominantly diurnal but day-long active snail *Physa acuta *[42].

However, other factors could be involved that may supersede daily activity rhythms as mediators in interactions between planarias and other benthic invertebrates. For example, predominantly nocturnal and carnivorous leeches and planarias naturally coexisting in Welsh lakes are well separated by differences in trophic behavior, with leeches acting as active predators and planarias behaving more like scavengers [35]. Also, visually hunting and hence diurnal odonate nymphs preferentially prey upon mobile organisms including *D. tigrina *[57], suggesting that time-independent chemo- and/or mechanoreception play(s) an important role in odonate predation. Since planarias themselves rely heavily on chemo- and mechanoreception [36,52], temporal partitioning may be only a cofactor of as yet unknown importance in mediating planarian-predator and dugesiid-gastropod interactions. As many aspects remain poorly understood despite a half-century-old research effort on dugesiid-gastropod interactions, comprehensive studies that would incorporate diel activity cycles are needed to fully understand the mechanisms and extent of dugesiid predation on snails.

Intra- and interspecific competition are primary factors regulating planarian populations and assemblages [18-20,24]. The relatively high activity (Figure 1) and quick response to day- and nighttime food inputs displayed by *D. polychroa *(Figure 3) may be associated with a continuous demand for energy, supporting the view that *D. polychroa *has high *per capita *energy investment and inherent poor competitive abilities [58]. Typical absence in unproductive waters but consistent presence — often in high numbers — in productive habitats [e.g., [26]] support this hypothesis, and further suggest that *D. polychroa*'s high activity and behavioral flexibility may compensate for poor competitive abilities when resources are plentiful. *D. tigrina*'s rigid nocturnal "window of opportunity" for hunting (Figures 1 and 3), which would limit access to prey, and successful colonization in productive but not nutrient-poor habitats as a nonnative invader [[17,26,27]; authors' personal observations], similarly suggest that *D. tigrina *also is a poor competitor *sensu latu*.

Initial coexistence between *D. tigrina *and native European planarias typically followed by replacement by *D. tigrina *[26,27], and absence of coexistence at high numbers in established communities [17,23,28], suggest that *D. tigrina *may not be as poor a competitor as *D. polychroa*. The high overlap in physicochemical requirements (e.g., preference for productive, hardwater lentic habitats: [22]), similar trophic spectra [16,17,21,33], and general nocturnal habits (this study) support the view that habitat-scale mutual exclusion between these two dugesiids is competition-driven [16-19,24,29,53].

Indeed, the "explosive" increase in the Colemere (UK) *D. tigrina *population in the 1980s and the concomitant decrease in the coexisting populations of *D. polychroa *and other native triclads [26] strongly suggest the involvement of interspecific competition as a regulating factor, as does the mutual exclusion of *D. polychroa *and *D. tigrina *in over 85% of the known local cases in mainland Britain (original elaboration of the data in [26]). Habitat-scale mutual exclusion between *D. polychroa/lugubris *and *D. tigrina *has been observed also in Italian lakes [[23,28]; authors' personal observations] and in Toronto Harbor (Ontario, Canada), where the there nonnative *D. polychroa *has been studied in detail [18,29]. Such an asymmetrical competition also supports the view that *D. polychroa*'s apparent specialization on gastropod prey is a niche refuge [e.g., [16]]. However, differences in habitat preference, with *D. polychroa *typically found at hard-bottom, well-lit sites [e.g., [27,29]], and *D. tigrina *seemingly preferring vegetated, shaded habitats [[23]; authors' personal observations], also may be involved. Whether such an apparent difference in habitat preference is related to *D. polychroa*'s higher tolerance of light irradiance, as our diel data suggest, is worth of further, *ad hoc *testing.

If competition is indeed behind the apparent mutual exclusion of *D. polychroa *and *D. tigrina*, their fine-scale temporal partitioning of habitat use (Figures 1 and 2) may not be sufficient to allow coexistence in the wild, in the same way that the broad, albeit nonsignificant, differences in daily peak activity between *D. tigrina *and *P. tenuis *(Figure 2), which also share much of their trophic spectra [17], may not preclude the *in situ *displacement of *P. tenuis *by colonizing *D. tigrina *[17,26]. However, as competition is virtually impossible to discern from mensurative observations [50,59], manipulative experiments specifically targeting this issue are needed to verify these hypotheses.

## Conclusions

The tested species are mainly nocturnal, consistent with their photonegative characteristics. However, only *D. tigrina *displayed strictly nocturnal habits. The predominantly nocturnal *D. polychroa *is active all day, potentially leading to more feeding opportunities but also higher predation risk. *P. tenuis*'s low degree of individual activity, unresponsiveness to food inputs, and late-night activity peak exhibited in the experiment may be related to a strong reliance on chemical and/or mechanical stimuli from coexisting planarias.

The fine-scale differences in (predominantly) nocturnal habits among these three triclad species, which also greatly overlap in habitat and trophic requirements, may not be sufficient to allow coexistence in the wild, with the nonnative *D. tigrina *eventually displacing the otherwise common *D. polychroa *and *P. tenuis *in many benthic communities in Europe.

Species-specific differences in circadian rhythms and other behavioral patterns are worthwhile of further, targeted investigations to aid in the understanding of interspecific interactions and distribution patterns of lake triclads.

## Competing interests

The authors declare that they have no competing interests.

## Authors' contributions

All authors participated in the development of the initial idea, the experimental design, and other conceptual aspects. PL, MG and FPM collected planarias *in situ *and managed the laboratory cultures. PL carried out the 10-d observations, analyzed the data statistically, and prepared the manuscript. PL, FPM and BC contributed to the discussion of the data, while MG's contribution was limited by his year-long illness and eventual passing during manuscript preparation. All surviving authors read and approved the final manuscript.

## Authors' information

PL is an adjunct research scientist at the University of L'Aquila (UoLA) and an independent environmental consultant based in Rome, Italy; her specialty are basic and applied aspects of shallow-water ecological communities, water quality issues, and applied limnology (lake management). MG was a laboratory manager and a full-time research scientist and FPM is an adjunct research scientist at UoLA; specialty areas for both were/are basic and applied aspects of benthic macroinvertebrate communities with an emphasis on water mite ecology. BC is the paper senior author and is full professor of ecology at UoLA and the UoLA representative in the nationwide Inter-University Consortium for Environmental Sciences and in the Board of Directors of the Sirente-Velino Regional Natural Park; he is specialized on water mite ecology, biodiversity, macroinvertebrate-based bioindication, and water quality issues in lotic systems.
